# Extensive Transcription Analysis of the *Hyposoter didymator* Ichnovirus Genome in Permissive and Non-Permissive Lepidopteran Host Species

**DOI:** 10.1371/journal.pone.0104072

**Published:** 2014-08-12

**Authors:** Tristan Dorémus, François Cousserans, Gabor Gyapay, Véronique Jouan, Patricia Milano, Eric Wajnberg, Isabelle Darboux, Fernando Luis Cônsoli, Anne-Nathalie Volkoff

**Affiliations:** 1 INRA - Université de Montpellier 2, Unité « Diversité, Génomes et Interactions Insectes-Microorganismes », Place Eugène Bataillon, CC101, Montpellier, France; 2 France Génomique - Commissariat à l'Energie Atomique - Institut de Génomique, Génoscope, 2, Evry, France; 3 Escola Superior de Agricultura Luiz de Queiroz - Universidade de Sao Paulo, Departamento de Entomologia e Acarologia, Laboratório de Interações em Insetos, Piracicaba, Sao Paulo, Brazil; 4 INRA - CNRS - Université Nice Sophia Antipolis, Institut Sophia Agrobiotech, Sophia Antipolis, France; University of Poitiers, France

## Abstract

Ichnoviruses are large dsDNA viruses that belong to the Polydnaviridae family. They are specifically associated with endoparasitic wasps of the family Ichneumonidae and essential for host parasitization by these wasps. We sequenced the *Hyposoter didymator* Ichnovirus (HdIV) encapsidated genome for further analysis of the transcription pattern of the entire set of HdIV genes following the parasitization of four different lepidopteran host species. The HdIV genome was found to consist of at least 50 circular dsDNA molecules, carrying 135 genes, 98 of which formed 18 gene families. The HdIV genome had general features typical of Ichnovirus (IV) genomes and closely resembled that of the IV carried by *Hyposoter fugitivus*. Subsequent transcriptomic analysis with Illumina technology during the course of *Spodoptera frugiperda* parasitization led to the identification of a small subset of less than 30 genes with high RPKM values in permissive hosts, consisting with these genes encoding crucial virulence proteins. Comparisons of HdIV expression profiles between host species revealed differences in transcript levels for given HdIV genes between two permissive hosts, *S. frugiperda* and *Pseudoplusia includens*. However, we found no evident intrafamily gene-specific transcription pattern consistent with the presence of multigenic families within IV genomes reflecting an ability of the wasps concerned to exploit different host species. Interestingly, in two non-permissive hosts, *Mamestra brassiccae* and *Anticarsia gemmatalis* (most of the parasitoid eggs were eliminated by the host cellular immune response), HdIV genes were generally less strongly transcribed than in permissive hosts. This suggests that successful parasitism is dependent on the expression of given HdIV genes exceeding a particular threshold value. These results raise questions about the mecanisms involved in regulating IV gene expression according to the nature of the lepidopteran host species encountered.

## Introduction

Polydnaviruses (PDV) are large circular dsDNA viruses carried by thousands of endoparasitic wasp species. Their genome is segmented into tens of molecules of various sizes. They are required for the successful development of the endoparasitic wasp and are thus commonly referred to as “viral symbionts”. The PDV genome is transmitted vertically, via a proviral form that persists in all wasp cells. PDV particles are produced from the proviral template exclusively in a specific tissue of the ovaries in the female, the calyx. They are stored within the lumen of the oviducts and are then injected into the insect host during oviposition [1]–[3]. Following parasitization, PDV particles rapidly infect most of the host cells, in which the genes encoded by the encapsidated viral genome are expressed. The expression of PDV genes modifies the host immune response and host development, favoring the successful development of the parasitoid within its host [4]–[10]. PDVs do not replicate in the parasitized insect, as the genes required for replication are present in the wasp genome and are not packaged into the virus particles [11],[12].

PDVs are classically separated into Bracoviruses (BVs), carried by endoparasitoid species from the Braconidae Microgastroid complex, and Ichnoviruses (IVs), carried by two subfamilies of ichneumonid wasps, the Campopleginae and the Banchinae [11]. BVs and IVs have different origins, and this has resulted in differences in the morphology of their virions, the nature of the genes encoding the particle proteins and the nature of the genes carried by the encapsidated genome. Only 10 PDV genomes have been entirely sequenced to date from among the PDVs carried by about 40,000 wasp species: five BVs, carried by *Cotesia congregata* (CcBV), *Microplitis demolitor* (MdBV), *Glyptapanteles flavicoxis* (GfBV), *Glyptapanteles indiensis* (GiBV) and *Cotesia vestalis* (CsBV) [12]–[16]; three IVs carried by the campoplegines *Campoletis sonorensis* (CsIV), *Hyposoter fugitivus* (HfIV) and *Tranosema rostrale* (TrIV); and two IVs carried by the banchines *Glypta fumiferanae* (GfIV) and *Apophua simplicipes* (AsIV) [11],[13],[17],[18]. The sequencing of PDV genomes has revealed a number of general conserved features: all PDVs have large genomes (190 to 610 kb), with a low coding capacity (15–35%), more similar to eukaryotic genomes than to classical viral genomes, and a large proportion of the genes are organized into multigene families. Indeed, most of the 61 to 197 genes identified in the various PDV species can be grouped into four to 13 gene families (reviewed in [19]). Only a small subset of the genes identified in PDV genomes display similarity to eukaryotic genes of known function, such as the BV protein tyrosine phosphatases (*PTP*), and the IV viral innexin genes, or the viral ankyrin genes found in all PDVs sequenced to date. A large proportion of the other genes identified in PDVs display no similarity to known sequences and their exact function remains unknown. Nevertheless, it is now widely accepted that PDV encapsidated genomes mostly contain genes encoding effectors essential for successful parasitism and probably derived from insect genes (i.e. those of the wasp) (reviewed in [19].

The issues of the presence of numerous multigene families in PDV genomes and their maintenance during evolution remain largely unexplored. It has often been suggested that the genome organizations of these viruses, which probably result from genome duplication events [11]–[14],[17],[20], and reviewed in [19], allows them to maintain a set of genes encoding proteins with additive or complementary roles in parasitism. As PDVs do not replicate in the cells of the parasitized insect, the presence of multigene families may facilitate an indirect increase in effector protein production, increasing viral efficiency. However, gene duplication may also lead to the paralogs acquiring new functions or mechanisms of gene regulation, due to mutations in their coding or non-coding sequences. PDV gene organization into multigene families may, therefore, also increase viral efficiency by allowing the virus to target different host tissues, physiological functions or signaling pathways. Indeed, it has been shown that members of multigene families may display different patterns of transcription in different host tissues [21]–[24]. Another little explored hypothesis is that gene duplication and the probable neo-functionalization of PDV genes may favor the adaptation of endoparasitoids to new insect hosts. For instance, the genomes of PDVs associated with parasitoids attacking a small number of host species tend to contain fewer genes than those of PDVs associated with species parasitizing a large number of host species [17].

One of the first steps towards determining the role of PDV genome organization in the adaptation of the parasitic wasp to its host species is analysis of the transcription of all the genes encoded by the PDV genome during the parasitization of various insect host species. Only a few such studies have been carried out to date, and all have focused on a single host species. Only one complete transcriptome analysis has been performed to date, for MdBV, which contains a small number of genes that have been analyzed by qPCR [21]. The same technique has also been used to follow the transcription of a subset of genes encoded by TrIV during parasitization [25]. Indirect data have also been obtained for the *Diadegma semiclausum* IV (DsIV), in a study involving RNAseq analysis of the parasitized host transcriptome [26]. All these studies have shown differences in PDV transcript levels between host tissues or between the members of a given multigene family.

We sequenced the circular dsDNA composing the *Hyposoter didymator* encapsidated Ichnovirus (HdIV) genome and studied its transcription. We found that the genome of HdIV, the fourth genome of an IV carried by a campoplegine wasp to be sequenced, had general features typical of IV genomes, with a high degree of similarity to the genome of HfIV, the IV carried by the related species *Hyposoter fugitivus*. *Hyposoter didymator* can parasitize a number of species from the Noctuidae family. We carried out analyses of the whole HdIV transcriptome with Illumina technology, following of the parasitization of two permissive and two non-permissive lepidopteran host species. Oviposition occurred in all four host species but the parasitoid was eliminated by the host cellular immune response in non-permissive hosts. A comparison of the HdIV transcriptomes between the two permissive hosts revealed that (1) a subset of viral genes consistently generated larger numbers of transcripts than the other genes. This subset included a number of genes now known to be specific to HdIV, suggesting that they may encode proteins essential for successful parasitism, and (2) transcript levels were up- or downregulated, depending on the lepidopteran host, although this concerned only a few HdIV genes. Finally, the transcriptome study showed that most of the HdIV genes produced smaller numbers of transcripts in non-permissive than in permissive hosts, suggesting that there may be a minimum level of HdIV gene expression for successful parasitoid development.

## Materials and Methods

### Insect origin, rearing and parasitism

Four lepidopteran species were used in bioassays: *Spodoptera frugiperda*, *Mamestra brassicae*, *Pseudoplusia includens* and *Anticarsia gemmatalis*. All originated from laboratory colonies (*S. frugiperda* was derived from the DGIMI laboratory colony, *P. includens* and *A. gemmatalis* from the “Interações em Insetos » laboratory colonies and *M. brassicae* was provided by E. Jacquin-Joly, INRA-France). *S. frugiperda* and *M. brassicae* were maintained on a semi-synthetic maize diet, whereas *P. includens* and *A. gemmatalis* were maintained on a semi-synthetic bean-based diet, all under the same stable conditions (24±2°C; 75–65% relative humidity; 16 h light: 8 h dark photoperiod). The wasp *Hyposoter didymator* used in the experiments was derived from the DGIMI laboratory colony, reared on *S. frugiperda* at 26±2°C with a 16 h light: 8 h dark photoperiod.

For the bioassays, early third-instar caterpillars of similar weight (between 2 and 7 mg, depending on the host species) were individually introduced into a glass vial containing 10 two-day-old female *H. didymator* wasps. The host larvae were removed immediately after they had been stung, and they were maintained in an incubator at 24±2°C; 75–65% relative humidity; 16 h light: 8 h dark photoperiod for the rest of the experiment. We selected 10 parasitized caterpillars at random from the parasitized pool and dissected them to check for the presence of an egg, to ensure that oviposition had occurred.

### Changes in host physiology induced by parasitization

We estimated the efficiency of parasitization and the rate of encapsulation by dissecting a batch of parasitized hosts 12–36 hours post-parasitism (p.p.) and recording the egg status of *H. didymator* (no egg, free egg or encapsulated eggs) for each of the hosts dissected. Another batch of hosts was dissected 72 hours p.p., for determination of the larval status (no larva, free larva or encapsulated larva) of each of the hosts dissected. The numbers of larvae dissected 12–36 p.p. and 72 h p.p. were 38 and 18, respectively, for *S. frugiperda*, 38 and 20 for *P. includens*, 54 and 61 for *M. brassicae* and 42 and 36 for *A. gemmatalis*.

We investigated the effect of parasitization on host weight gain, by weighing the parasitized caterpillars individually at 6 hours, 72 hours and 6 days p.p. The numbers of larvae (control/parasitized) used were 35/42 for *S. frugiperda*, 24/40 for *P. includens*, 37/35 for *M. brassicae* and 32/56 for *A. gemmatalis*.

### Preparation and sequencing of HdIV genomic DNA

Genomic DNA was extracted from HdIV as previously described [27] from filter-purified HdIV particles collected from 300 dissected ovaries. The extracted HdIV dsDNA was then sequenced at the Génoscope, with the same shotgun and Sangers sequencing strategies used for the CcBV encapsidated genome, as previously described [12]. The sequences obtained were assembled with PHRED and PHRAP, as previously described [12]. In some cases, segment sequence assembly was further validated by comparing the segment sequence obtained with that of *H. didymator* genomic clones, when such sequences containing the corresponding proviral sequence were available, using a private BAC library available in our laboratory (Volkoff *et al.*, unpub.).

### HdIV segment analysis

We used the Blastn algorithm (http://www.ncbi.nlm.nih.gov/BLAST/) [28] to compare all of the identified HdIV segments, to assess potential sequence similarities between segments. We then checked for sequence similarity to segments of other IVs (TrIV, HfIV and CsIV) by using the Blastn algorithm to search the NCBI public nucleotide database. Synteny between the HdIV and HfIV segments was visualized with the ARTEMIS Comparison Tool (ACT) interface (www.sanger.ac.uk). Protein sequences were aligned with the ClustalW online tool [29].

### RNA isolation and sequencing

For the analysis of HdIV transcripts in different lepidopteran species, we extracted total RNA from pools of five caterpillars, with the RNeasy purification kit (Qiagen). RNA was extracted 6 hours p.p. for the four lepidopteran host species, and then 24 hours and 72 hours p.p. for *S. frugiperda*. RNA samples were treated with the Turbo DNAse-free Kit (Ambion) and we checked that there was no contaminating DNA, by PCR with specific primers binding to the HdIV P30_Hd6 intron and the *S. frugiperda* ELF-1 (elongation-like factor 1) exon. Total RNA concentration was estimated with a Nanodrop ND-1000 spectrophotometer. Total RNA quality was assessed by electrophoresis in a 1% agarose gel and with an Agilent 2100 bioanalyzer (Agilent Technologies, Santa Clara, California). For each host species and/or set of conditions, we carried out two or three independent extractions. Sequencing with Illumina technology (Hi-Seq 2000; 50 bp single reads) was then performed either by the GATC company (www.gatc-biotech.com/fr/) (3 samples, collected 6 hours p.p. for each of *S. frugiperda*, *P. includens*, *M. brassicae* and *A. gemmatalis*, and 1 sample each obtained at 24 and 72 hours p.p. for *S. frugiperda*) or by the MGX CNRS public sequencing facility (www.mgx.cnrs.fr/) (2 samples for *S. frugiperda*, obtained at 24 and 72 hours p.p.). More than 11 million 50 bp-long single reads were obtained for each sample (Table S1).

### HdIV genome annotation, read mapping and RPKM counting

For each HdIV genome segment, the open reading frames (ORF) present were predicted with the ARTEMIS interface (www.sanger.ac.uk) [30]. Only ORFs encoding proteins of more than 99 amino acids (a.a.), with an initiator methionine, were initially considered as putative genes. In cases of overlapping ORFs, we considered only the ORF displaying sequence similarity to known PDV genes or with limits consistent with RNAseq mapping (see below) data. For all putative genes, we carried out public database searches for sequence similarities with the NCBI Blastn, Blastx and tBlastx algorithms (http://www.ncbi.nlm.nih.gov/BLAST/) [28]. Searches for specific regulatory motifs in the upstream gene region were performed with the SCOPE and MEME online tools.

For all RNA samples sequenced, the corresponding raw data Tag-sorted FastQ Files containing the adaptor-cleaned single reads (between 47 and 50 bp/read) were used for mapping procedures on the 135 HdIV genes in BOWTIE [31], with classical parameters: a –v 1 –best –sam. Using the ARTEMIS interface, we determined the number of reads that could be mapped for all annotated HdIV genes. For each gene, the RPKM was calculated as defined by [32] using the following formula RPKM = 10^9^ *C/NL* where C is the number of reads that could be mapped to gene exons, N is the total number of mappable reads in the experiment, and L is the sum of the lengths of the exons (in base pairs). We avoided RPKM overestimation for genes containing perfectly repeated sequences longer than 47 bp (*i.e.* the read length), by obtaining the final RPKM for these genes by dividing the read counts by the number of repeats identified within the gene sequence. We checked that all the reads mapped with HdIV were actually viral genes, by also mapping reads acquired from unparasitized *S. frugiperda* larval samples. None of the reads for this sample mapped onto HdIV genes, demonstrating the absence of sequence similarity between *S. frugiperda* and HdIV genes. We estimated a background threshold, by calculating the RPKM corresponding to the intergenic regions of each HdIV segment from the reads acquired for the *S. frugiperda* 72 h p.p. sample. The RPKM value obtained for HdIV intergenic regions was consistently below 0.03 (this may have been due to slight contamination of the RNA samples with HdIV DNA despite the DNAse treatment). As a consequence, we considered only HdIV genes with an RPKM value >0.1 to be effectively transcribed.

### Statistical analysis

We compared the frequencies of encapsulated *H. didymator* eggs and larvae between the four hosts dissected at 12–36 h and 72 h p.p., by logistic regression analysis with a generalized linear model especially designed for the modeling of binomial data, using a logit link function to compare mean values [33]. For the weight gain of host larvae, we used a simple one-way ANOVA to compare the mean values obtained between parasitized and control (non-parasitized) larvae, at 6 h, 72 h and 6 d p.p.

For HdIV transcriptome analysis, we then clustered HdIV genes statistically into classes defined on the basis of their level of transcription during parasitization (classes A, B and C for high, medium and low RPKM levels, respectively), using the automatic Bayesian classification system available from the AutoClass@IJM webserver (http://ytat2.ijm.univ-paris-diderot.fr/) [34]. For the analysis of transcription during the parasitization of *S. frugiperda*, we used a two-way ANOVA to detect significant differences in transcription levels (RPKM values) for the HdIV genes, by analyzing the difference (as a fold-change) between the reference sample (mean value of *S. frugiperda* 6 h p.p.) and the other two sets of conditions tested (*S. frugiperda* 24 and 72 h p.p.). Finally, a two-way ANOVA was used to detect significant differences in the transcription level of each HdIV gene between the four host species (*S. frugiperda* 6 h p.p., *P. includens* 6 h p.p., *M. brassicae* 6 h p.p. and *A. gemmatalis* 6 h p.p.).

## Results and Discussion

### General features of the HdIV encapsidated genome

**Figure 1:**
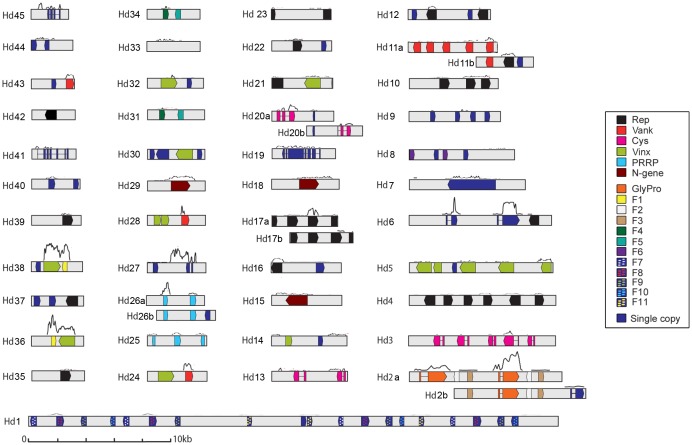
Graphic representation of the HdIV genome and transcribed regions. The 50 HdIV segments are shown in linear form, from the smallest (Hd45, 2548 bp) to the largest (Hd1, 36006 bp). Pairs of HdIV segments with similar sequences were designated, the two segments of each pair being named a and b. Colored boxes show the open reading frames (ORFs). Refer to the legend for color correspondences. Introns within ORFs are indicated by a line between two colored boxes (corresponding to exons). A “transcript coverage curve” is shown above each HdIV segment (i.e. the number of Illumina reads mapping to the segment sequence; for the sake of simplicity, only data from one of the 3 “72 h p.p.” replicates were used to draw the curve). Provisional GenBank accession numbers: Hd45:KJ586284; Hd44:KJ586285; Hd43:KJ586286; Hd42:KJ586287; Hd41:KJ586288; Hd40:KJ586289; Hd39: KJ586290; Hd38:KJ586291; Hd37:KJ586292; Hd36:KJ586293; Hd35:KJ586294; Hd34:KJ586295; Hd33: KJ586296; Hd32:KJ586298, Hd31:KJ586299; Hd30:KJ586300; Hd29:KJ586303; Hd28:KJ586304; Hd27:KJ586305; Hd26b:KJ586306; Hd26a:KJ586301; Hd25:KJ586307; Hd24:KJ586308; Hd23:KJ586309; Hd22:KJ586310; Hd21:KJ586311; Hd20b:KJ586297; Hd20a:KJ586312; Hd19:KJ586313; Hd18:KJ586315; Hd17b:KJ586316; Hd17a:KJ586314; Hd16:KJ586317; Hd15:KJ586318; Hd14:KJ586319; Hd13:KJ586320; Hd12:KJ586321; Hd11b:KJ586302; Hd11a:KJ586322; Hd10:KJ586323; Hd9:KJ586324; Hd8:KJ586325; Hd7:KJ586326; Hd6:KJ586328; Hd5:KJ586329; Hd4:KJ586330; Hd3:KJ586331; Hd2b:KJ586327; Hd2a:KJ586332; Hd1:KJ586333.

The main characteristics of the sequenced HdIV encapsidated genome, presented in Table 1, are very similar to those reported for other IV genomes (reviewed in [19]): 50 circular molecules ranging in size from 36 kb (segment Hd1) to 2.5 kb (segment Hd45), a genome size of 263 kb, a GC content of 43%, and 134 putative ORFs, corresponding to 31% of the genome (Figure 1; Table S2).

**Table 1:** General features of the HdIV, HfIV, TrIV and CsIV genomes.

	HdIV	HfIV	TrIV	CsIV
Genome size (Kb)	263	246	176	247
GC content (%)	43	43	42	41
Segment number	45+5	56	20	22
Putative ORF number	134 (+1**)	150 (+4*)	89	101
Multigene families members	98	77	39	58
*Cys*	*9*	*5*	*1*	*10*
*N-gene*	*3*	*3*	*4*	*2*
*PRRP*	*5*	*11*	*1*	*5*
*Rep*	*26*	*38*	*17*	*30*
*Vank*	*8*	*9*	*2*	*7*
*Vinx*	*14*	*11*	*3*	*4*
*TrV*	*/*	*/*	*7*	*/*
*OSSP*	*/*	*/*	*4*	*/*
*F0 (GlyPro)*	*2 (+*1**)	*/*	*/*	*/*
*F1*	*2*	*1*	*/*	*/*
*F2*	*2*	*/*	*/*	*/*
*F3*	*2*	*/*	*/*	*/*
*F4*	*2*	*/*	*/*	*/*
*F5*	*2*	*1*	*/*	*/*
*F6*	*2*	*/*	*/*	*/*
*F7*	*4*	*/*	*/*	*/*
*F8*	*4*	*1*	*/*	*/*
*F9*	*4*	*/*	*/*	*/*
*F10*	*3*	*/*	*/*	*/*
*F11*	*3*	*/*	*/*	*/*
Single-copy genes	37	70 (+4*)	50	43
Putative genome coding density	30%	30%	22%	29%

For each of the 4 sequenced IVs, we show: the genome size, the calculated genome % GC content, the number of known viral segments, the total number of putative open reading frames (ORF), the number of ORFs belonging to a multigene family, the number of ORFs currently identified for each of the multigene families (see text for a description of the families; OSSP: ovary-specific secreted protein), the number of ORFs present as single copies, and the calculated predicted IV genome coding density. (+1*) indicates the additional HdIV gene previously described and not identified during sequencing in this study; (+4**) indicates the 4 new HfIV ORFs identified in this work by alignment with HdIV sequences (see Figure S1).

Interestingly, 10 HdIV segments contained identical regions of variable length (Figure 1). These common regions were probably derived from a common proviral sequence, –as demonstrated in some cases by sequence comparison with *H. didymator* BAC clones (data not shown). These 10 HdIV segments were grouped into five pairs of sequence, with the members of each pair differentiated by “a” and “b” designations (Hd2a/Hd2b with 6976 identical bp in common, Hd11a/Hd11b with 1394 identical bp, Hd17a/Hd17b with 3655 identical bp, Hd20a/Hd20b with 902 identical bp and Hd26a/Hd26b with 2878 identical bp). Consequently, in cases in which the overlapping region contained an open reading frame (ORF), the gene was present twice in the encapsidated form of the HdIV genome (Figure S1). Finally, sequencing also revealed that 11 of the HdIV segments (Hd1, Hd2ab, Hd3, Hd4, Hd5, Hd6, Hd7, Hd10 and Hd26ab) contained at least two internal direct repeat sequences (Table S2), suggesting possible nesting, as previously described for CsIV [17],[35],[36].

In total, the encapsidated HdIV genome contains at least 135 genes: 134 ORFs predicted from the sequenced viral genome, plus a previously described HdIV gene (*GlyPro3* GenBank #AF132023.1) [22],[27],[37]–[40] not identified in the newly acquired HdIV sequence. The absence of this sequence indicates that at least one HdIV segment was not sequenced in this study, for unknown reasons. This finding raises questions about how we can ensure that the entire genome is sequenced in the case of complex genomes, such as those of PDVS (e.g. multipartite, with a high frequency of repeated regions). Wasp genome sequencing data (e.g. as the wasp genome contains the proviral form) will undoubtedly provide the answer to this question in the future.

We found that 65 of the total of 135 predicted HdIV genes could be grouped into the six multigene families classically found in Campopleginae IV genomes [13],[17]: the “Cysteine motif proteins” (Cys), “Repeat element” (Rep), “Viral innexins” (Vinx), “Viral ankyrins” (Vank), “Polar-Residue-Rich Proteins” (PRRP) and “N-genes” families (Table 1). In HdIV, the most abundant gene families are the *Rep* family (26 genes), as for other Campopleginae IVs, and the *Vinx* family (14 genes). In addition, the HdIV genome contains 12 other gene families encoding proteins of unknown function (Table 1). These families have been named *HdIV_F0* (corresponding to the previously described *GlyPro* family [27]) to *HdIV_F11*, and each of these families contains two to four gene copies (Table 1). Lastly, HdIV genome contains 37 single-copy genes (Table 1), including four that have already been described (*D8_Hd50* (GenBank #AF464931.1), *K19_Hd29* (GenBank #AF241775.1), *SerThr_Hd7* (*S6 mRNA*, GenBank #AF464930.1) and *P30_Hd6* (Orf1, GenBank #AF479654.1)) [38],[40]. Nineteen of the 135 HdIV genes contained at least one intron (Figure 1), a classical characteristic of PDV genes. These genes included the 9 *Cys* genes, the 3 members of the *GlyPro* family, *P30_Hd6*, *U1_Hd6*, *SerThr_Hd7*, *D8_Hd45*, *K19_Hd29*, *U1_Hd41* and *U1_Hd19*. As reported in previous studies [27],[38], the first exon, corresponding to a putative signal peptide, was found to be conserved in several of these genes (*GlyPro* family, *P30_Hd6*, *U1_Hd6* and *SerThr_Hd7*).

Blastn similarity searches against the NCBI public database revealed strong similarities between HdIV and the IV carried by the wasp *Hyposoter fugitivus*, HfIV: 44 of the 50 HdIV segments displayed nucleotide sequence identity to 42 of the 56 HfIV segments (Table S2), in their gene or intergenic regions. By contrast, except for genes belonging to multigene families conserved in IV, no significant sequence similarity was found to segments from other sequenced IV genomes. This conservation of the viral segment sequences within a given wasp genus, with differences probably related to diversification of the wasp genus, has also been described for bracoviruses [14].

Interestingly, comparisons between HdIV and HfIV led to the identification of four new coding sequences in HfIV, one in each of the 4 HfIV segments C1, C8, C15 and C17 (named *U1_HfC1*, *U1_HfC8*, *U1_HfC15* and *U1_HfC17* indicated in Figure S2, A). Portions of the intergenic regions of the HdIV and HfIV segments were very similar (more than 65% nucleotide identity), probably reflecting a common ancestral origin for the segments. The newly identified HfIV genes each had two exons, the first of which presented a high degree of sequence similarity to the first exon of the abovementioned HdIV genes (Figure S2, B). This first-exon sequence is thus conserved in diverse IV genes carried by *Hyposoter* wasps, possibly due to a currently unknown mechanism for the *de novo* acquisition of signal peptides. Conversely, the second exon was found to be divergent both within and between species, suggesting a possible divergence of the functions of the corresponding proteins. Such divergence between the two *Hyposoter* species may reflect the adaptation of the two wasp species to different host ranges. Indeed, *H. didymator* is known to parasitize mostly lepidopteran species from family Noctuidae and a few species from the Pieridae, Nymphalidae and Lasiocampidae families [41], whereas *H. fugitivus* mostly parasitizes species from the Lasiocampidae, Arctiidae, Saturniidae, Notodontidae and Lymantriidae families [42].

Thus, the known HdIV encapsidated genome contains 37 single-copy genes and 98 genes clustered into 18 gene families: 65 belong to the six families conserved in all IVs, and the other 33 belong to 12 other families named *HdIV_F0* to *HdIV_F11* (Table 1). One member of each of the HdIV_*F1*, *F5* and *F8* families was identified in HfIV, and one member of the HdIV_*F1* family was identified among the DsIV RNAseq contigs. Sequence comparisons between HdIV and other known IV genes also indicated that 10 single-copy HdIV genes displayed sequence similarity to HfIV and one (*K19_Hd27*) displayed sequence similarity to DsIV (Table S2). Thus, 9 HdIV gene families and 28 single-copy genes have been described only in HdIV, suggesting that at least some of these genes and gene families were acquired more recently in the *Hyposoter* lineage.

### Analysis of HdIV transcription during *S. frugiperda* parasitization

Recent studies on the interactions of *H. didymator* with its permissive host *S. frugiperda* have shown that HdIV infection is essential for successful parasitism, particularly for the early larval stages of the parasitoid (48 h–72 h p.p.) [43]. The availability of the HdIV genome sequence made it possible to determine which HdIV genes were transcribed in the insect host and, potentially, to identify those involved in inducing the changes to host physiology required for parasitoid development. We identified HdIV genes expressed during the parasitization of *S. frugiperda*, by analyzing the HdIV transcriptome in whole host larvae and at three stages of parasitism, corresponding to the embryonic (6 h and 24 h p.p.) and early larval (72 h p.p.) development stages of *H. didymator*.

**Figure 2:**
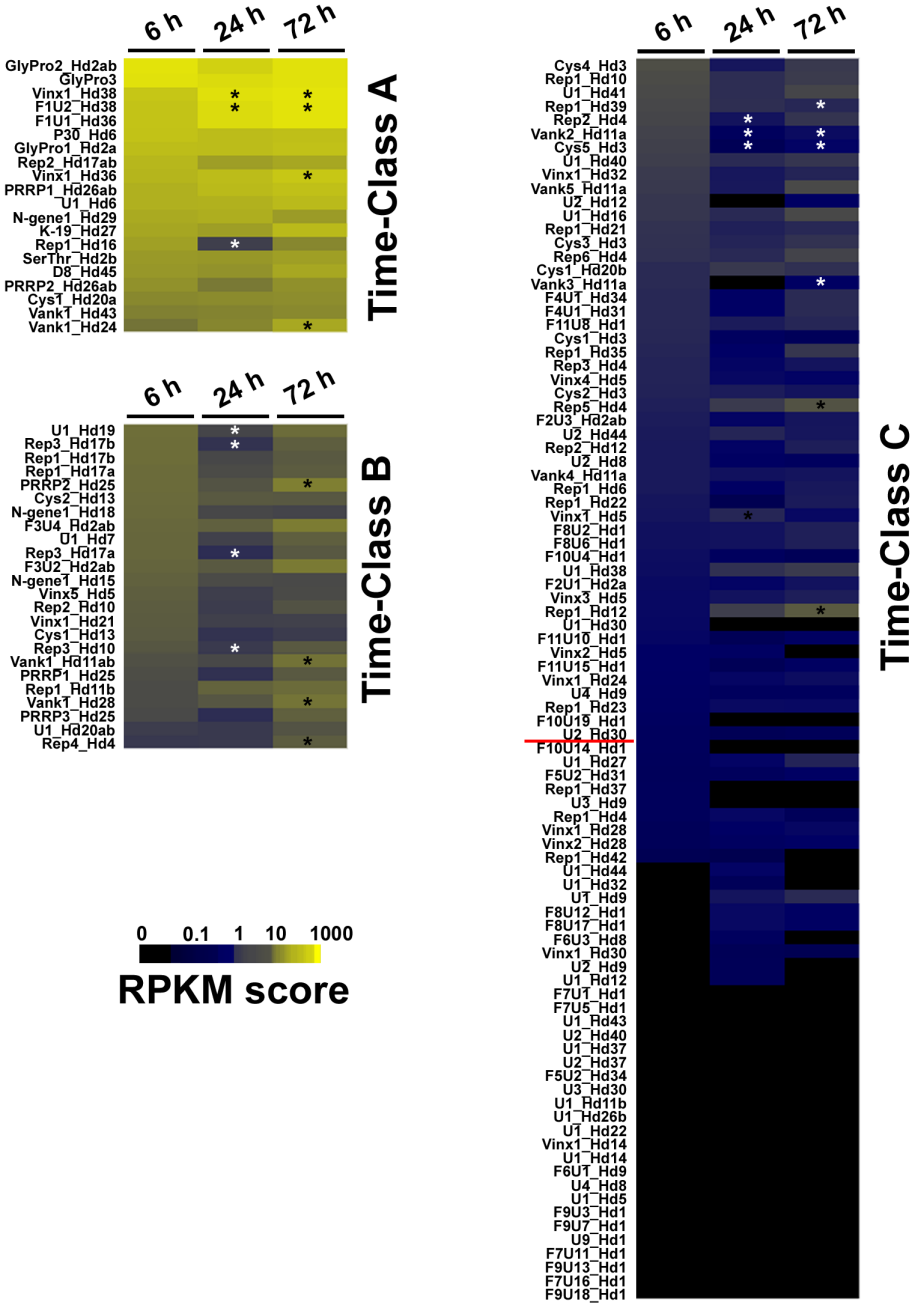
Profile of HdIV transcript levels during the time-course of *S. frugiperda* parasitization. Each row represents the mean RPKM value for each of the 135 HdIV genes analyzed in *S. frugiperda* at 6 h, 24 h and 72 h p.p. The color scale (black to yellow) indicates RPKM level. The RPKM values were used to cluster genes into classes (A, B and C) with the AutoClass algorithm available from the AutoClass@IJM website. Asterisks indicate significant (*p*>0.05) RPKM fold-changes with respect to the arbitrary chosen reference (*Sf* 6 h p.p. sample). White asterisks indicate a significant decrease and black asterisks indicate a significant increase in transcript levels (fold-changes ranging from 2 to 67).

We used an automatic Bayesian classification system [34] based on mean RPKM values and taking the three sampling periods into account, to classify the 135 HdIV genes into three classes (A (RPKM>18), B (3<RPKM<18) and C (RPKM<3)) as a function of their level of transcription during parasitization (Figure 2). Class A (RPKM>18) contained only 20 of the 135 HdIV genes predicted by *in silico* analyses (Figure 2, Time-Class A). Five of the seven genes with the highest RPKM values, were intron-containing genes specific to *H. didymator* (*P30_Hd6*, the 3 members of the *GlyPro* family and *U1_Hd6*). RNAseq thus confirmed previous results concerning transcription obtained by the less sensitive northern-blot method for the three members of the *GlyPro* family and *P30_Hd6* [38],[39]. All five HdIV-specific genes were found to encode secreted proteins characterized by repeated amino-acid motifs (glycine- and proline-rich for the *GlyPro* family, serine- and threonine-rich for *P30*_Hd6), but their function remains unknown [27],[38]. Class A also included at least one gene from each of the six conserved IV multigene families (2 *Rep*, 2 *Vank*, 2 *Vinx*, 1 *Cys-motif*, 2 *PRRP* and 1 *N-gene*). Our results thus also confirm previous descriptions of high transcript levels for *Rep2_Hd17ab*, *Rep1_Hd16*, *Vank1_Hd24* and *Vank1_Hd43* obtained with RT-qPCR techniques [22],[37] and for *SerThr_Hd2b*, *K19_Hd27* and *D8_Hd45* by northern-blot analyses [27],[38]. The homologs of several HdIV Class A genes (*vank1_Hd24, Vinx1_Hd38, PRRP1_Hd26ab, N-gene_Hd29, K19_Hd27* and *F1U2_Hd38*) were also the genes with the highest levels of transcription in the indirect study of DsIV gene transcription during *Plutella xylostella* parasitization by *D. semiclausum* (DsIV vankyrin 1 (GenBank #JI257593), viral innexin 1 (GenBank #JI257597), polar residue-rich protein (GenBank #JI257606), unknown protein (GenBank #JI257608), unknown protein (GenBank #JI257609), and unknown protein (GenBank #JI257611) genes) [26]. Class B (3<RPKM<18) contained 24 HdIV genes (Figure 2, Time-Class B) and included at least one member of each of the six multigene families conserved in IVs (2 Vank, 2 Vinx, 8 Rep, 2 Cys-motif, 3 PRRP, 2 N-gene genes), and members of the *Hd_F3* and *Hd_F6* families, together with two single-copy genes (*U1_Hd7* and *U1_Hd19*). Class C (RPKM<3) contained 91 HdIV genes (Figure 2, Time-Class C). However, based on the RPKM threshold for background (determined for HdIV segment intergenic regions, see Materials & Methods), only 50 HdIV genes were considered to be truly transcribed (RPKM>0. 1) in parasitized *S. frugiperda* larvae. However, we cannot exclude the possibility that the transcription of the remaining 41 genes was not detected because they are actually transcribed in specific host tissues and their transcripts are thus rare in analyses of RNA from the entire larva. Alternatively, they may be transcribed exclusively in other insect host species.

Our results clearly indicate that a small subset of HdIV genes are more strongly transcribed than the others. This difference may be accounted for by a corresponding larger number of gene copies within the viral particles, and delivered to the caterpillar. However, not all the genes present in a given HdIV molecule had a high RPKM (for instance *Vank1_Hd24* in class A and *Vinx1_Hd24* in class C; Figure 2), indicating that the differences between HdIV transcript levels cannot be accounted for entirely by differences in segment molarity. We investigated these observed differences further, by searching for regulatory motifs in the upstream regions of a subset of genes with RPKM values greater than 10 (24 genes present in 19 segments). However, this analysis revealed no particular characteristic upstream sequences linking high levels of expression with a given regulatory mechanism.

The time-course analysis of the HdIV transcriptome during *S. frugiperda* parasitization showed that, for most of the HdIV genes, transcript levels were already high 6 h p.p. Cell infection and HdIV transcription are therefore rapid in *S. frugiperda* larvae. Nevertheless, increases or decreases in the levels of transcripts for some genes were observed over the time course (at 24 and 72 h p.p. versus the reference time point 6 h p.p.) (Figure 2). Such changes were observed for the genes from the Hd38 and Hd36 segments (*F1U1_Hd36* and *F1U2_Hd38* for the most transcribed), several *Vank* genes (*Vank1_Hd11ab*, *Vank1_Hd28* and *Vank1_Hd24*), *PRRP2_Hd25* and *Rep4_Hd4*, which were expressed more strongly at 24 or 72 h p.p. than at 6 h p.p. Conversely, *Rep1_Hd16*, *U1_Hd19* and *Rep3_Hd17ab* were less strongly expressed at 24 h p.p. than at 6 h p.p. Such changes in transcript level during the course of parasitization may indicate, for these particular HdIV genes, the existence of transcriptional regulation by host cellular/development factors.

The highly transcribed genes, particularly those mentioned above as being conserved in the Campopleginae lineage (on the basis of their sequence and transcription pattern), may be essential for changes in host physiology successful parasitism. However, our knowledge of the role of the corresponding proteins remains limited, essentially because these proteins display no similarity to any known protein of known function. For those belonging to multigene families conserved in IVs, the related proteins have been studied in other biological models and the available data suggest that many play a role in immunosuppression. For instance, the CsIV viral innexins can form functional GAP junction hemi-channels and thus interfere with cell-cell interactions in the lepidopteran host, particularly during the encapsulation process [44]. Thus, HdIV *Vinx* (*Vinx1_Hd38* and *Vinx1_Hd36*), the transcript levels of which increased during the parasitization time course (Figure 2, Time-Class A), may be involved in disruption of the encapsulation process, as previously observed in parasitized *S. frugiperda* [9]. Similarly, the Cys1_Hd20a protein may be involved in alterations to cellular immunity. Effectively, two secreted CsIV cysteine-motif proteins, VHv1.1 and VHv1.4, decrease host hemocyte cell adhesion capacity by an unknown mechanism [45]. The HdIV transcriptome analysis also showed that four *Vank* genes (*Vank1_Hd24*, *Vank1_Hd43*, *Vank1_Hd28* and *Vank1_Hd12ab*) were highly transcribed during parasitism. The *Vank* family is present in all PDVs (BV and IV), suggesting that the corresponding proteins are essential for parasitism success (reviewed by [46]). Experiments *in vitro* and in cells have shown that BV vankyrins interact with the insect host transcription factor NF-kB and that, like the IkB protein, they maintain NF-kB in its inactive form [47],[48]. Signaling through NF-kB regulates diverse cellular and physiological processes that could consequently be affected by vankyrins. For instance, two CsIV Vanks expressed in a heterologous *in vivo Drosophila* system have been shown to impair the cellular immune response, humoral inflammation and embryonic development of *Drosophila*, with phenotypes similar to those observed in NF-kB-deficient flies (*Dorsal* and *Dif* (encoding NF-kB factors) silenced (RNAi) or mutant *Drosophila*) [49]. These previous investigations in the *Drosophila* model (which is not naturally parasitized by PDV-associated wasps) have highlighted the potential function of the Vanks, but these findings require validation in natural lepidopteran models. Some IV Vanks are also known to inhibit lepidopteran cell apoptosis [50],[51]. By preventing apoptosis of the IV infected cell, IV Vanks may ensure the maintenance of the non-replicative IV genome and gene transcription throughout parasitism. Additional *in vivo* investigations of the function of HdIV proteins are required to understand the as yet unknown role of these proteins in parasitism, and our results suggest that proteins encoded by genes abundantly transcribed in the *S. frugiperda* host should be given priority, although other genes are probably also required for successful parasitism.

### Assessment of the permissiveness of different lepidopteran host species

Analysis of the HdIV transcriptome during the parasitization of *S. frugiperda* showed that less than one third of the HdIV genes had high RPKM values. This raises questions about the importance of the remaining HdIV genes, particularly those with RPKM values below the threshold of 0.1. *H. didymator* can develop in several noctuid species. It is therefore possible that HdIV genes are differentially transcribed in different host species. We tested this hypothesis, by determining HdIV transcript levels after the parasitization of permissive and non-permissive lepidopteran hosts.

We first assessed the permissiveness of different host species to *H. didymator* development. We assessed oviposition by *H. didymator* in *S. frugiperda* and three other species of noctuids: *P. includens*, *M. brassicae* and *A. gemmatalis*. We found that the females readily laid eggs in larvae of all these species but that only *S. frugiperda* and *P. includens* larvae were permissive for the development of *H. didymator* offspring (wasp pupae obtained for more than 70% of the parasitized hosts).

**Figure 3:**
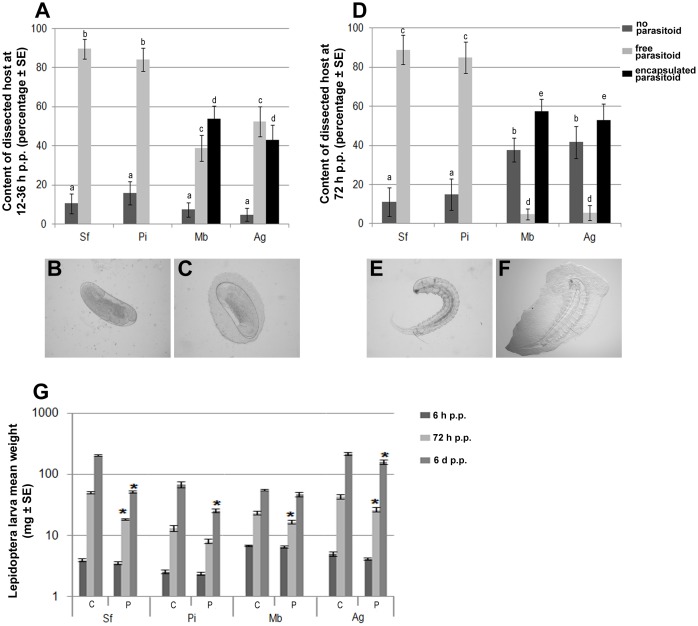
Encapsulation response to parasitization by *H. didymator* in four lepidopteran host species. **A**. Percentage of larvae from which *H. didymator* eggs were recovered following host dissection 12–36 h p.p. **B. and C**. Examples of *H. didymator* eggs recovered between 12–36 h p.p from a permissive host (*S. frugiperda* or *P. includens*) and a non-permissive host (*M. brassicae* or *A. gemmatalis*), respectively. **D**. Percentage of *H. didymator* larvae recovered following host dissection 72 h p.p. **E. and F**. Example of an *H. didymator* larva recovered at 72 h p.p. from a permissive host and a non-permissive host, respectively. Note that, in **C**. and **F**., a layer of host immune cells surrounds the parasitoid; this is known as encapsulation. In **A**. and **D**., different lowercase letters indicate significantly different (*p*<0.05) results. **G**. Weight of lepidopteran larvae at 3 times p.p. depending on the species. C: control host larvae, P: host larvae parasitized by *H. didymator*. Asterisks indicate that, for one species and one time, body weight differed significantly between control and parasitized hosts (*p*<0.05). Sf: *S. frugiperda*, Pi: *P. includens*, Mb: M. *brassicae* and Ag: *A. gemmatalis*.

We assessed the permissiveness of the different host species further, by evaluating several host physiological traits known to be affected by HdIV. The first of the traits measured evaluated the efficiency of the host immune response, through measurement of the *H. didymator* encapsulation rate at various time points after parasitism (12 h, 36 h and 72 h p.p.). As expected, neither *S. frugiperda* nor *P. includens* could encapsulate the parasitoid eggs or larvae (Figure 3 A, B, D and E). Conversely, more than 40% of the parasitoid eggs recovered from *M. brassicae* and *A. gemmatalis* were encapsulated (Figure 3 A and C), and more than 80% of the parasitoid larvae that escaped encapsulation at the egg stage were also found to be encapsulated (Figure 3 D and F). The second trait measured was used to evaluate the impact of parasitization on host growth rate. Indeed, previous studies have shown that HdIV plays a major role in the lower weight gain observed in parasitized *S. frugiperda* larvae than in control larvae [9]. As for *S. frugiperda*, parasitized *P. includens* larvae gain significantly less weight than non-parasitized larvae (Figure 3 G). However, in *P. includens*, this difference was smaller and occurred later (Figure 3 G). Parasitized *A. gemmatalis* displayed a significantly smaller weight gain than controls 3 and 6 days p.p. For *M. brassicae*, a significant decrease in growth rate was observed only 3 days p.p. (Figure 3 G).

These data confirm that *P. includens* is a permissive host, whereas *M. brassicae* and *A. gemmatalis* are both non-permissive species. Our results indicate that, under laboratory conditions, the main barrier to successful *H. didymator* parasitism on *M. brassicae* and *A. gemmatalis* is the host cellular immune response. We previously showed that, in *S. frugiperda*, the egg is protected against encapsulation by as yet unidentified surface proteins, whereas HdIV is involved in the immune protection of the parasitoid larvae [43]. In the case of *M. brassicae* and *A. gemmatalis*, none of these effectors are effective, resulting in high encapsulation rates for both eggs and parasitoid larvae. *H. didymator* uses a combination of virulence strategies to escape the host immune system [43], so future investigations will be required to investigate the effects on each of these strategies in non-permissive hosts. However, the observed lower weight gain in parasitized two non-permissive hosts strongly suggests that these species are nevertheless affected by HdIV, the main source of virulence factors in the *S. frugiperda*/*H. didymator* interaction [9].

### Comparative analysis of HdIV transcription in different lepidopteran host species

We analyzed the profile of HdIV gene transcription following the parasitization of *P. includens*, *M. brassicae* and *A. gemmatalis*. The transcription profiles in these different lepidopteran host species were then compared. No major variations in HdIV transcription profile over time were observed in the time analysis of *S. frugiperda* parasitism, so all transcriptome analyses were conducted at 6 h p.p.

Illumina sequencing resulted in the detection of HdIV transcripts in all the parasitized host species, with 97, 56 and 69 HdIV genes having RPKM values >0.1 for *P. includens*, *M. brassicae* and *A. gemmatalis*, respectively (88 genes in *S. frugiperda* 6 h p.p.). Thus, HdIV particles can infect cells from different lepidopteran species but this infection is not necessarily associated with successful parasitoid development. HdIV infection may account for the significant decreases in weight gain on parasitization observed for the non-permissive hosts.

**Figure 4:**
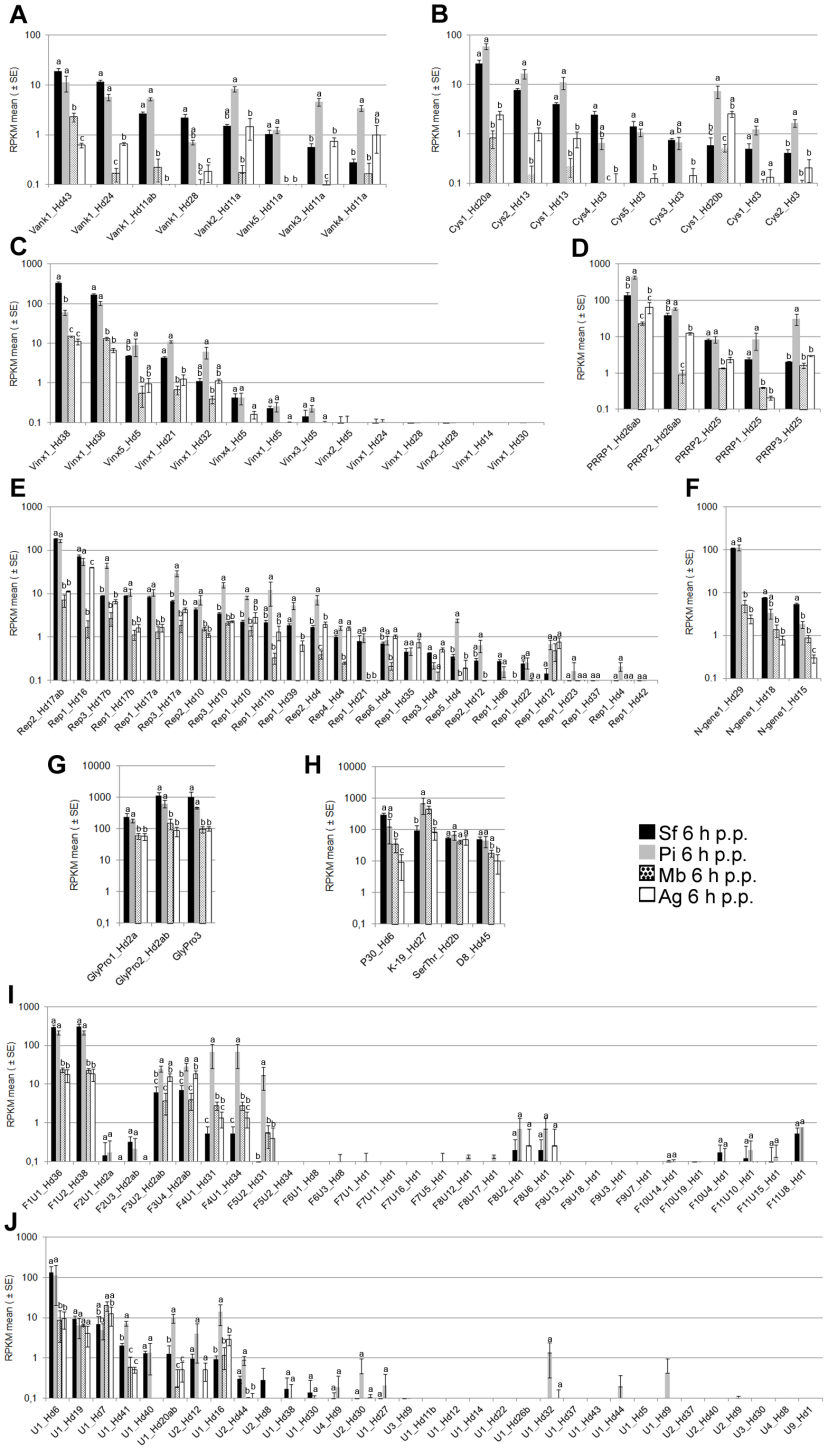
The HdIV transcriptome in different lepidopteran host species (6 h p.p.). RPKM levels are indicated for genes belonging to the *Vank* family (**A**), the *Cys-motif* family (**B**), the *Vinx* family (**C**), the *PRRP* family (**D**), the *Rep* family (**E**), the *N-gene* family (**F**), the *GlyPro* family (**G)**, the known single-copy gene (**H**), the F1 to F11 family (**I**) and the newly characterized single-copy gene (**J**). For each HdIV gene, different lowercase letters indicate a significant difference (*p*<0.01).

A comparison of two permissive hosts, *P. includens* and *S. frugiperda*, confirmed our unexpected finding that most HdIV genes were expressed at very low levels (or apparently not at all) and led us to draw three main conclusions: (i) in general, the genes for which transcripts are the most abundant in *S. frugiperda* are also the most strongly transcribed in *P. includens*; (ii) there are significant differences in gene transcript levels between the two permissive host species, but only for a small number of genes; (iii) these observed differences did not necessarily correspond to different genes from the same multigene family (i.e. none of the observed increases in expression for a particular gene was correlated with a decrease in expression for another gene from the same gene family). Thus, most HdIV genes produced similar amounts of transcript in the two permissive hosts, *P. includens* and *S. frugiperda* (Figure 4, Sf and Pi). Nevertheless, significant differences in transcript levels were observed for a number of genes. For instance, *Vank3&Vank4_Hd11a*, *Cys1_Hd20b*, *Cys1_Hd13, PRRP3_Hd25*, Rep3_Hd17b, Rep3_Hd17a, Rep3_Hd10, Rep2_Hd4, Rep5_Hd5, K-19_Hd27, F3U2_Hd2ab, F3U4_Hd2ab, F4U1_Hd31, F4U1_Hd34, F5U2_Hd31, U1_Hd41, U1_Hd20ab and U1_Hd16 had significantly higher RPKM values in *P. includens* than in *S. frugiperda*. Conversely, a few HdIV genes, such as *Vinx1_Hd38* and *Ngene1_Hd15* in particular, generated fewer transcripts in *P. includens* than in *S. frugiperda*. Overall, the data obtained suggest that some of the global physiological changes observed in the two permissive hosts may be mediated by different viral effectors. However, too little is currently known about the function of HdIV proteins to test this hypothesis. Finally, we observed no major variation or inversion of patterns of RPKM values within any of the HdIV multigene families as a function of host species (Figure 4, Sf and Pi). Thus, based on this analysis of only two permissive lepidopteran species, there seems to be no clear relationship between the existence of multigene families in HdIV and the possibility of the associated parasitoid being able to exploit a large range of host species.

The above observations for the comparison of the two permissive hosts also applied to the comparison of the two non-permissive hosts *M. brassicae* and *A. gemmatalis* (transcription levels generally higher in *A. gemmatalis* regardless of the gene considered; Figure 4 Mb and Ag). However, a comparison of permissive and non-permissive hosts showed that HdIV gene transcription rates were much lower in the non-permissive hosts (generally by a factor of log10; Figure 4). In the non-permissive hosts tested 6 h p.p., a smaller number of HdIV genes were found to be transcribed and overall transcript levels were lower than those in permissive hosts (Figure 4). Relevant examples are provided by *Cys1_Hd20a*, *PRRP2_Hd26ab* and *Rep1_Hd16*, which had RPKM values below 2 in *M. brassicae* but over 20 in the permissive hosts. One explanation for the failure of parasitism in *M. brassicae* and *A. gemmatalis* is that low transcript levels result in protein levels that are too low to induce all the changes in host physiology required for the successful development of the parasitoid larva. There may be several reasons for the observed lower levels of transcripts in the non-permissive hosts at early stages of parasitism. Firstly, assuming that *H. didymator* females inject the same volume of calyx fluid during oviposition, regardless of the size of the host, the density of viral particles injected into the host may be lower in non-permissive hosts, as *M. brassicae* and *A. gemmatalis* are slightly larger than the two permissive hosts (Figure 3G). Alternatively, HdIV particles may infect the host cells of non-permissive species less efficiently, either because they penetrate a smaller number of cells or because they are eliminated by an antiviral immune response mediated by autophagy or apoptotic mechanisms, as already described in insects [52]–[54]. A third, non-mutually exclusive, alternative is that putative host regulatory factors (enhancers) are absent from these species.

Our high-throughput transcriptome results complement existing knowledge acquired through experiments on the wasp *C. sonorensis*, which showed only that non-permissive hosts were not able to sustain CsIV infection during parasitism [55],[56]. Based on this previous work, we could assume that the low level of HdIV gene transcription measured at 6 h p.p. in the two non-permissive hosts (*M. brassicae* and *A. gemmatalis*) in our system may decrease still further during parasitization, accounting for the recovery and normal development of non-permissive hosts (Figure 3G, Mb and Ag 6 d p.p.) and efficient immune responses directed against wasp larvae escaping encapsulation (Figure 3D, Mb and Ag).

One of the key conclusions of this study is that, despite the variations of expression observed for a subset of HdIV genes, the global HdIV gene transcription profile does not differ between parasitized host species. This may be because there is no need for a diversification of the molecular mechanisms driving HdIV gene expression to allow *H. didymator* to exploit a large range of host species. However, our study concerned only a limited number of *H. didymator* hosts, and many other lepidopteran host species need be analyzed before any firm conclusions can be drawn.

## Conclusion

This study generated the fourth campoplegine IV annotated genome sequence to be published and is, to our knowledge, the first global time-course transcriptome analysis of an IV during parasitization and on different lepidopteran host species. The general features of the HdIV genome are similar to those of the genomes of other IVs. Like other IVs, HdIV has a genome consisting of a large number of small circular DNA molecules (49 molecules of 2.5 to 8.9 kb in size, plus a large, 36 kb segment), and most of its 135 viral genes are organized into multigene families. We identified nine gene families and 28 single-copy genes currently unknown outside of HdIV, which may have been acquired more recently in the *Hyposoter* lineage.

The principal result of this study, that only a small proportion of HdIV genes are strongly transcribed in parasitized hosts, was unexpected. In an analysis of HdIV gene transcription in the entire parasitized insect host, only a subset (less than 30) of the HdIV genes, most harbored by only 19 of the 50 HdIV segments, was found to be strongly transcribed (RPKM>50) in the two permissive hosts, *S. frugiperda* and *P. includens*. This raises a number of questions:

Why these particular genes? The most plausible hypothesis is that these genes encode proteins required for successful parasitism. The “top10” genes in this list were all genes specific to HdIV (Figure 4 G, H) that appear to have been acquired recently, during the process of *H. didymator* speciation/adaptation to its host range. Thus, although transcript levels do not always reflect protein levels, these HdIV genes may be considered good candidates for future functional characterization, to improve our understanding of their role in parasitism and to explore epistasis within the HdIV genome. Our results also indicate that total HdIV gene transcript levels are low in non-permissive hosts. This may indicate that HdIV infection is “abnormal” (e.g. inefficient cell entry and/or gene expression) in these lepidopteran species, resulting in failed parasitism.

How is the expression of these genes regulated? The possible mechanisms involve gene-specific promoter sequences and/or a larger number of gene copies (within the virus particles) thanks to classical segment nesting or the existence of overlapping sequences (e.g. Hd2, Hd17, Hd26). Comparisons between different host species also indicated the probable involvement of host factors in regulating expression, because some genes are differentially expressed between host species, even though this is not generally the case. Our preliminary search for characteristics of the upstream sequences of abundantly transcribed genes was not conclusive, probably because the dataset available is still too limited. Finally, thanks to the apparent great plasticity of PDV genomes, mechanisms for the regulation of particular genes may have been acquired through duplication events and/or recombination between segments (e.g. Hd36 and Hd38, Hd24 and Hd28, Hd31 and Hd34, both encoding an F4 gene more strongly transcribed in *P. includens* than in *S. frugiperda*) or within segments (e.g. Hd6 and, to a lesser extent, Hd20).

Why are genes that are only weakly expressed or not expressed at all retained in the PDV genome? There are several possible answers to this question: (i) they may be “active” at very low levels and/or in very specific tissues or physiological/cellular processes of the host, or (ii) even if not strictly required, the proteins they encode may increase the chances of parasitism being successful (e.g. synergistically). In any case, it would be interesting to study these genes in detail, to determine the type of selection operating on them (e.g. neutral, positive), once the lack of data for other related biological models (sequences, transcription level) has been overcome. The work performed here dealt with entire host larvae and it is known that some IV genes are differentially transcribed between host tissues [25],[37],[57],[58]. We, therefore, cannot rule out the possibility that some of the genes producing small numbers of transcripts in the whole host organism are expressed strongly in specific host tissues. Future studies should therefore investigate the tissue specificity of HdIV gene expression, to evaluate the overall role of HdIV genes in parasitism.

Overall, the data presented here constitute a first, but crucial step towards understanding the global functioning of the HdIV genome during the parasitoid/host interaction.

## Supporting Information

Figure S1 **Graphic representation of the overlapping segment in proviral (a) and circularized form (b).** The Hd11a and Hd11b segments are illustrated. These two segments are integrated into the wasp chromosome in such a way that their ends overlap (a). During the circularization process (b), the DRJL and the DRJR (Direct Repeat Junction Left or Right) of each segment recombines to produce different segments with a common sequence.
(JPG)

Figure S2 **Sequence synteny between ichnovirus segments.** **A**. Regions of synteny between 2 HdIV (Hd2b and Hd6) and 4 HfIV (respectively C1 & C17 and C8 & C15) segments are shown in red (>65% nucleotide sequence identity). A “transcript coverage curve” is shown above each HdIV segment (i.e. number of Illumina reads mapping to the segment sequence; data from only one of the three “72 h p.p.” replicates were used to draw the curve). Colored boxes represent the HdIV and HfIV putative ORFs; refer to the legend for color correspondence. The newly annotated *U1_HfC1*, *U1_HfC8*, *U1_HfC15* and *U1_HfC17* HfIV ORFs are indicated. **B**. Amino-acid alignment of the regions corresponding to the first exon of the HdIV genes *SerThr_Hd2b*, *GlyPro1_Hd2a*, *GlyPro2_Hd2ab*, *P30_Hd6*, *U1_Hd6*, and the HfIV genes *U1_HfC1*, *U1_HfC8*, *U1_HfC15* and *U1_HfC17*.
(JPG)

Table S1 **Illumina sequence reads mapped onto the 135 HdIV genes for all conditions tested.** For each condition tested, the total number of Illumina reads (sequences) obtained is indicated, together with the number of reads mapping to each of the 135 HdIV genes.
(XLSX)

Table S2 **Characteristics of HdIV genome segments.** The features of the HdIV segments are indicated: name, size (nt), presence and position of internal direct repeats and Blastn sequence similarities to other IV segments (ID of the best blast, % identity, alignment length (nt), e-value). For each segment, the features of predicted ORFs are indicated: name, strand, position, length (nt) and blastx or tblastx sequence similarity to other IV genes (ID of the best blast, % identity, alignment length (nt), e-value).
(XLSX)
