# Supplementary figures and images for: Extensive Transcription Analysis of the Hyposoter didymator Ichnovirus Genome in Permissive and Non-Permissive Lepidopteran Host Species
Source: PLoS One. 2014 Aug 12;9(8):e104072. doi: 10.1371/journal.pone.0104072 (PMC4130501; doi:10.1371/journal.pone.0104072)

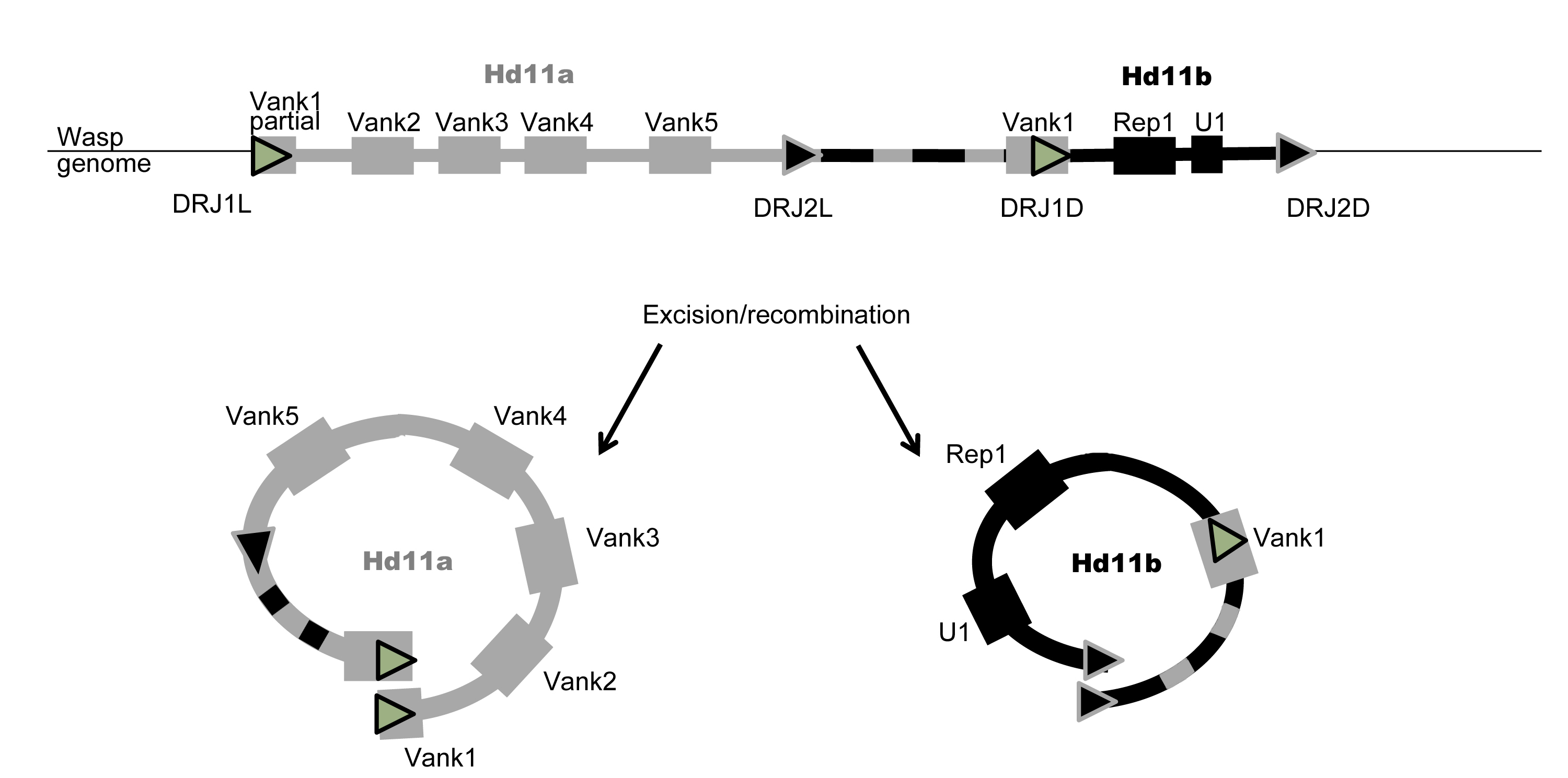

Supplement: Figure S1 — Graphic representation of the overlapping segment in proviral (a) and circularized form (b). The Hd11a and Hd11b segments are illustrated. These two segments are integrated into the wasp chromosome in such a way that their ends overlap (a). During the circularization process (b), the DRJL and the DRJR (Direct Repeat Junction Left or Right) of each segment recombines to produce different segments with a common sequence. (JPG) [file pone.0104072.s001.jpg]

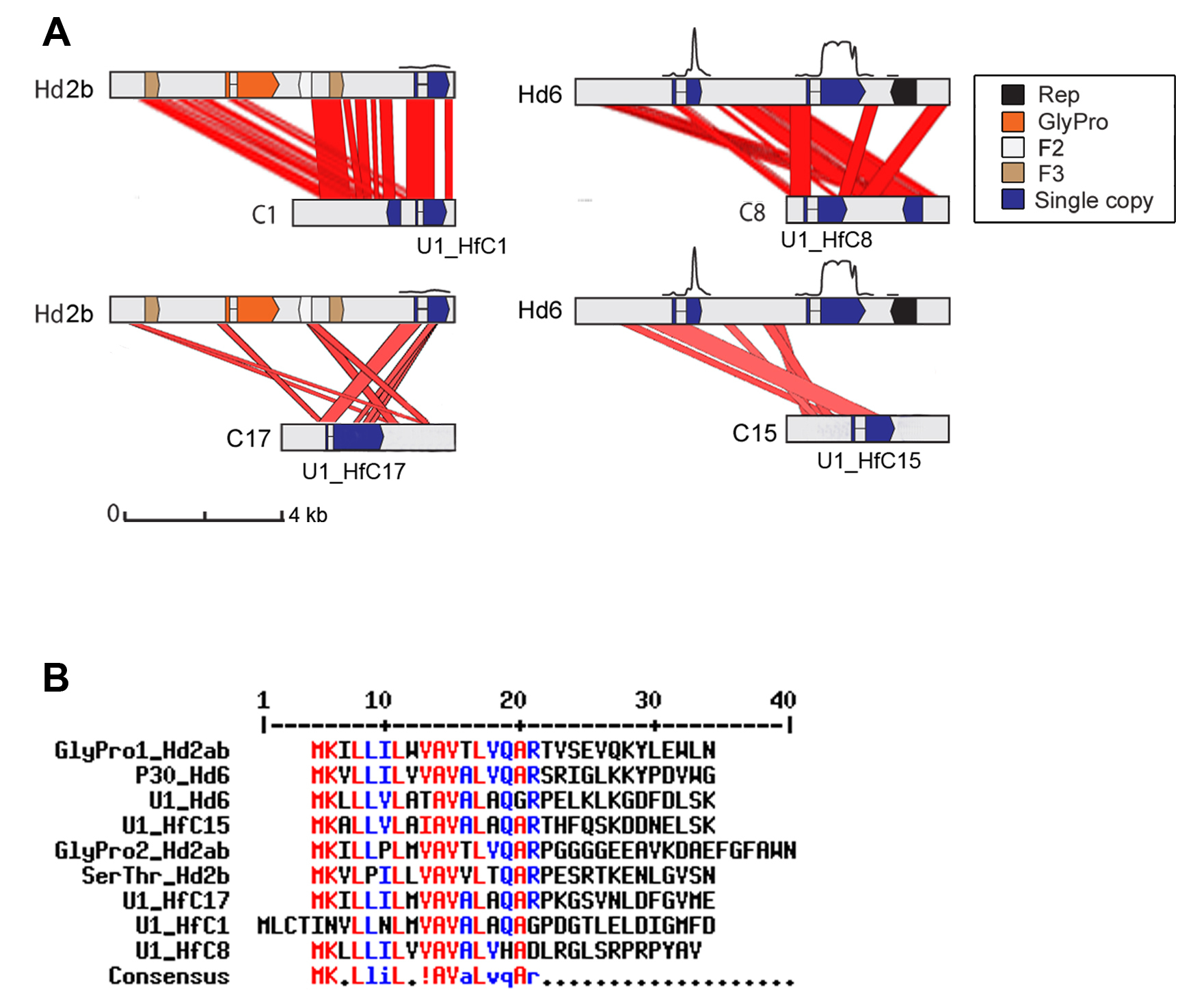

Supplement: Figure S2 — Sequence synteny between ichnovirus segments. A. Regions of synteny between 2 HdIV (Hd2b and Hd6) and 4 HfIV (respectively C1 & C17 and C8 & C15) segments are shown in red (>65% nucleotide sequence identity). A “transcript coverage curve” is shown above each HdIV segment (i.e. number of Illumina reads mapping to the segment sequence; data from only one of the three “72 h p.p.” replicates were used to draw the curve). Colored boxes represent the HdIV and HfIV putative ORFs; refer to the legend for color correspondence. The newly annotated U1_HfC1, U1_HfC8, U1_HfC15 and U1_HfC17 HfIV ORFs are indicated. B. Amino-acid alignment of the regions corresponding to the first exon of the HdIV genes SerThr_Hd2b, GlyPro1_Hd2a, GlyPro2_Hd2ab, P30_Hd6, U1_Hd6, and the HfIV genes U1_HfC1, U1_HfC8, U1_HfC15 and U1_HfC17. (JPG) [file pone.0104072.s002.jpg]
